# The propensity for re-triggered predation fear in a prey fish

**DOI:** 10.1038/s41598-020-65735-1

**Published:** 2020-06-09

**Authors:** Adam L. Crane, Laurence E. A. Feyten, Indar W. Ramnarine, Grant E. Brown

**Affiliations:** 1 Department of Biology, Concordia University, Montreal, Canada; 2 Department of Life Sciences, University of the West Indies, St. Augustine, Trinidad, Tobago

**Keywords:** Ecology, Psychology

## Abstract

Variation in predation risk can drive variation in fear intensity, the length of fear retention, and whether fear returns after waning. Using Trinidadian guppies, we assessed whether a low-level predation threat could easily re-trigger fear after waning. First, we show that background risk induced neophobia after either multiple exposures to a low-level threat or a single exposure to a high-level threat. However, a single exposure to the low-level threat had no such effect. The individuals that received multiple background exposures to the low-level threat retained their neophobic phenotype over an 8-day post-risk period, and this response was intensified by a single re-exposure to the low-level threat on day 7. In contrast, the neophobia following the single high-level threat waned over the 8-day period, but the single re-exposure to the low-level threat on day 7 re-triggered the neophobic phenotype. Thus, despite the single low-level exposure being insufficient to induce neophobia, it significantly elevated existing fear and re-triggered fear that had waned. We highlight how such patterns of fear acquisition, retention, and rapid re-triggering play an important role in animal ecology and evolution and outline parallels between the neophobic phenotype in fishes and dimensions of post-traumatic stress in humans.

## Introduction

The fear of predation is a unifying theme across vertebrate taxa^1^. Such reactions involve changes in neural morphology and physiology that promote behaviours such as increased vigilance, freezing, and neophobia (i.e., the fear of novelty)^2–4^. Natural selection should favour fear behaviours that allow prey to avoid predation (i.e., antipredator behaviours), but such defences can be energetically costly and decrease time available for foraging and other fitness-related behaviours^5,6^. Hence, prey should assess predation risk in their environment and balance trade-offs to optimize the intensity of their antipredator responses^6^. Indeed, numerous studies have documented that prey are sensitive to the level of threat posed by a predator, with prey showing increased fear behaviour in the presence of higher-level threats (i.e., ‘threat sensitivity’)^7,8^. Several studies have manipulated background predation risk by simulating situations where prey are vulnerable to attack or are exposed directly to cues from predator attacks^9^. Even after the predation threat is gone, fear behaviour can persist, as evidenced by studies that have conducted testing at different time points post risk^10–12^.

Cognitive research reveals that fear can wane through various mechanisms^13^. For instance, repeated exposures to risk can weaken fear, as animals become desensitized^14–16^. However, re-exposure to risk can also cause previously weakened or extinguished fear to re-emerge. This phenomenon has been widely observed among rats and humans and is often referred to as the ‘return-of-fear’ (or fear ‘renewal’)^17,18^. Such re-triggering of fear can be promoted by events before, during, or after risk exposure, and can depend on the temporal sequence of events. In humans, for instance, risk later in life often appears to re-trigger post-traumatic stress that has waned after being acquired from risk exposure earlier in life^19^. Moreover, the return of fear appears to be promoted by background risk in the form of multiple risk exposures that are combined into a short time period, rather than spaced apart^20^. In addition to the timing of risk exposure, the spatial context of risk exposure can promote the return of fear, as demonstrated by experiments where the testing context differed from the extinction context (i.e., the context where fear was lost)^21–23^.

For decades, fear behaviour has been studied in fishes. Many species have a substance in their skin, originally described as ‘Schreckstoff’ by von Frisch^24,25^, that is released into the water upon physical damage from a predator. Nearby conspecifics (and sometimes heterospecifics) can detect this substance via olfaction, recognize that an attack has occurred, and react with alarm^26,27^. Hence, these substances have become commonly referred to as ‘alarm cues’. Many species can use alarm cues to acquire predator recognition, a learning process that requires only a single exposure to alarm cues paired with the visual and/or chemical cues of a predator (i.e., a predator conditioning)^28,29^. Moreover, repeated exposure to alarm cues is known to induce phenotypically-plastic neophobia in fishes^30^. In cichlids, *Amatitlania nigrofasciata*, induced neophobia is sensitive to the intensity of background threat and persists longer following larger threats^31^. In fathead minnows, *Pimephales promelas*, induced neophobia can be more intense after experiencing risk in isolation, whereas risk experienced in a social group appears to cause neophobia that persists longer^32^. In Trinidadian guppies, *Poecilia reticulata*, there is evidence that fear is more intense and lasts longer following frequent exposure to brief threats^33^.

In some animal species, even a single exposure to predation risk has been found to induce a fearful state that can persist long after risk has ceased^34^. However, nearly all previous studies on induced neophobia via alarm cues have involved repeated exposures. To our knowledge, only one previous study has reported neophobia induced by a single alarm cue exposure^35^. In that study, rainbow darters, *Etheostoma caeruleum*, were conditioned with conspecific alarm cues paired with a novel odour. Subsequently, (2 d post risk), the darters showed a fear response to the conditioned odour, but also showed fear toward a novel control odour. In much of the older literature on alarm-cue learning, novel testing cues were not used and hence did not assess whether a single exposure induced neophobia. However, several more recent studies on generalization of predator-recognition learning have demonstrated that a single conditioning with alarm cues can induce learned responses to phylogenetically similar odours but not a broader fear response toward all novel stimuli^36,37^. We suspected that whether neophobia was induced by a single exposure in these studies might be explained by differences in the intensity of the threat (i.e., differences in alarm cue concentration). While the aforementioned studies each used ecologically relevant concentrations of alarm cues, the use of different species has involved different methodologies, with a seemingly higher concentration being used in the study finding induced neophobia^35^.

**Figure 1:**
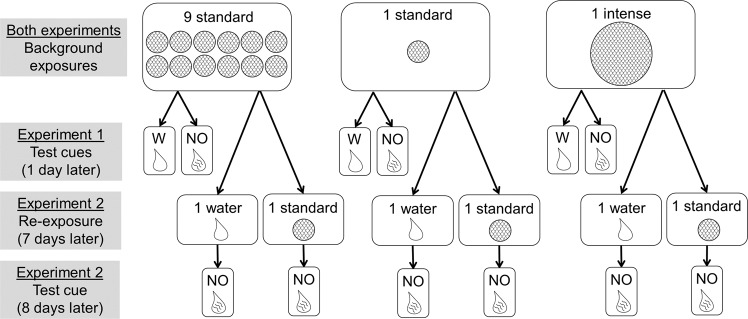
Experimental design. Guppies experienced background risk from exposure to alarm cues (circles of fish skin) either 9 times at a standard concentration, 1 time at a standard concentration, or 1 time at an intense concentration that was 9 times the standard concentration (see methods for exposure details). One day following the background risk, the behaviour of half of the guppies was measured before and after exposure to either water (W) or novel odour (NO). The other half of guppies received a second risk treatment 7 days after the background risk period, being exposed to either the standard concentration of alarm cues or water. These fish were then tested the following day, before and after exposure to NO.

We had three objectives in this study: (1) determine whether a single exposure to an intense concentration of alarm cues can induce neophobia in Trinidadian guppies, (2) assess the retention of such fear responses post risk, and (3) explore whether a low-level threat can re-trigger fear after waning. We conducted two experiments where all guppies were exposed to one of three background risk regimes (Fig. 1). One group received multiple exposures (n = 9) to alarm cues at a concentration that we considered as a ‘standard’ concentration (details in methods) because it approximately matched concentrations that elicited guppy antipredator responses in previous studies^38,39^. Moreover, at this concentration, repeated exposures have been shown to induce neophobia in guppies^40^, and thus, this treatment group served as a positive control. The other background risk regimes involved only a single exposure to alarm cues, one with the standard concentration and the other with an intense concentration (equal to the total amount of the multiple exposure treatment) (Fig. 1). Based on preliminary observations, we did not expect that a single exposure at the standard concentration would induce a neophobic response, and thus we expected this treatment group to serve as a negative control. However, we predicted that the intense exposure would result in an elevated fear response based on the aforementioned studies that involved only a single exposure, but we had no a priori expectation for how this treatment group would compare to the multiple exposure group.

One day after the background risk period, we assessed the fear behaviour of half of the guppies from each background risk group, conducting observations before and after exposure to a novel odour (or a water control) to assess baseline fear and neophobia, respectively (Fig. 1). The other half of the guppies remained untested and in the absence of risk for 7 days. Then, they received a single re-exposure to either the standard concentration of alarm cues or a water control (Fig. 1). On the following day, we observed the behaviour (dashing, freezing, pacing, calm swimming, and foraging) of each guppy and calculated an overall ‘fear index’ (see methods for details) before and after exposure to the novel odour (i.e., a test for baseline fear and a test for neophobia). This re-exposure treatment allowed us to determine whether neophobic responses were retained (water re-exposure group) and whether a low-level threat could re-trigger a fear response (alarm cue re-exposure group).

## Results

### Experiment 1: Initial fear

**Figure 2:**
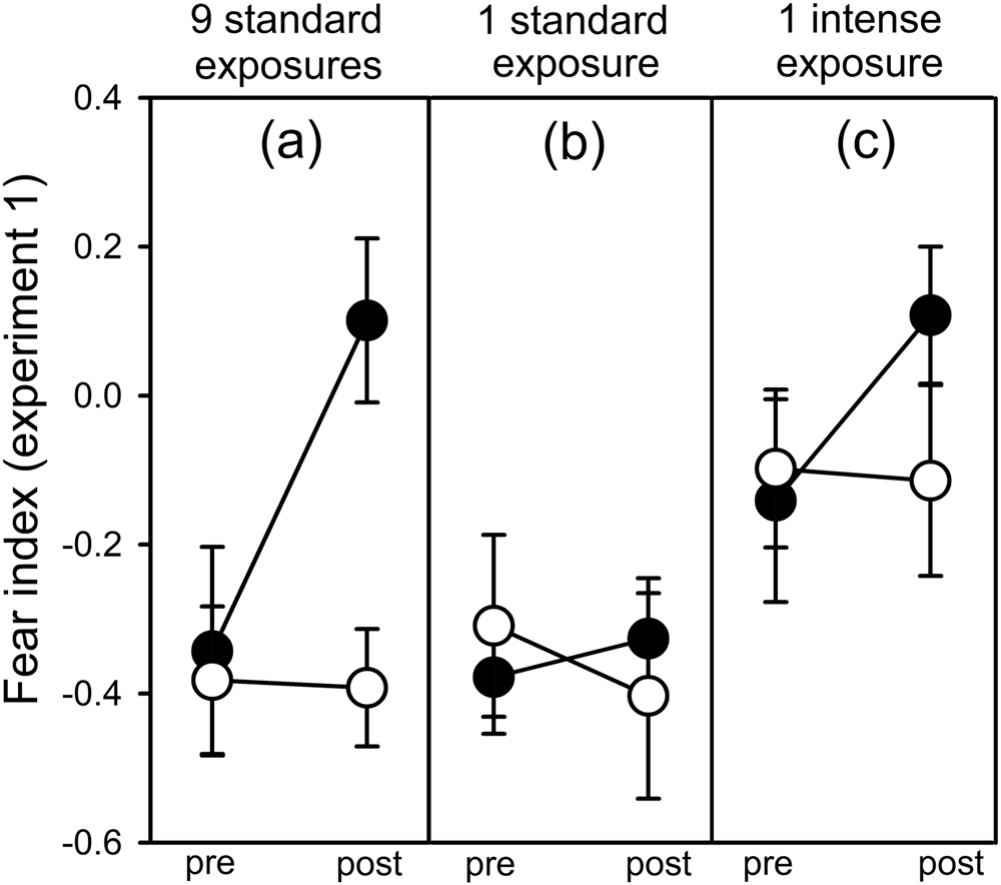
Mean (±SE) fear index from experiment 1 (testing initial fear). Guppies were tested before (pre) and after (post) injection of a novel odour (black circles) or water (white circles) 1 day after exposure to background predation risk via alarm cues [either 9 standard exposures (**a**), 1 standard exposure (**b**), or 1 intense exposure that was 9× higher than the standard exposure (**c**)]. See methods for exposure details.

In experiment 1, we found significant interactions involving each of the fixed factors (Table 1). Post-hoc testing revealed that the 9 standard exposures induced a significant neophobic response (time × test cue: *F*_1, 32_ = 14.80, *p* = 0.001; Table S1; Fig. 2a), whereas 1 standard exposure did not (time × test cue: *F*_1, 29_ = 3.20, *p* = 0.084; Table S1; Fig. 2b). In contrast, 1 intense exposure induced significant levels of both neophobia (time × test cue: *F*_1, 32_ = 4.47, *p* = 0.042; Table S1; Fig. 2c) and baseline fear (background risk: *F*_1, 13.4_ = 7.97, *p* = 0.014; Fig. 2c; Table S2).

**Table 1:** Overall GLMM for experiment 1.

	*F*	*df*	*p*
Time	13.69	1, 93	<0.001
Background risk	8.78	2, 18	0.002
Test cue	1.93	1, 80.7	0.17
Time × background risk	5.21	2, 93	**0.007**
Time × test cue	21.28	1, 93	**<0.001**
Background risk × test cue	0.75	2, 80.8	0.48
Time × background risk × test cue	2.27	2, 93	0.11
Background pail	0.41	21, 72	0.99
Subject	9.30	72, 93	<0.001

Output for testing the fixed effects of time (pre/post stimulus), the background risk treatment (9 standard exposures, 1  standard exposure, or 1 intense exposure), the test cue (novel odour or water), and their interactions on the fear index 1 day after the background risk treatment, with the background pail and the subject as random factors. Significant terms of interest are in bold type.

### Experiment 2: Re-triggering fear

**Figure 3:**
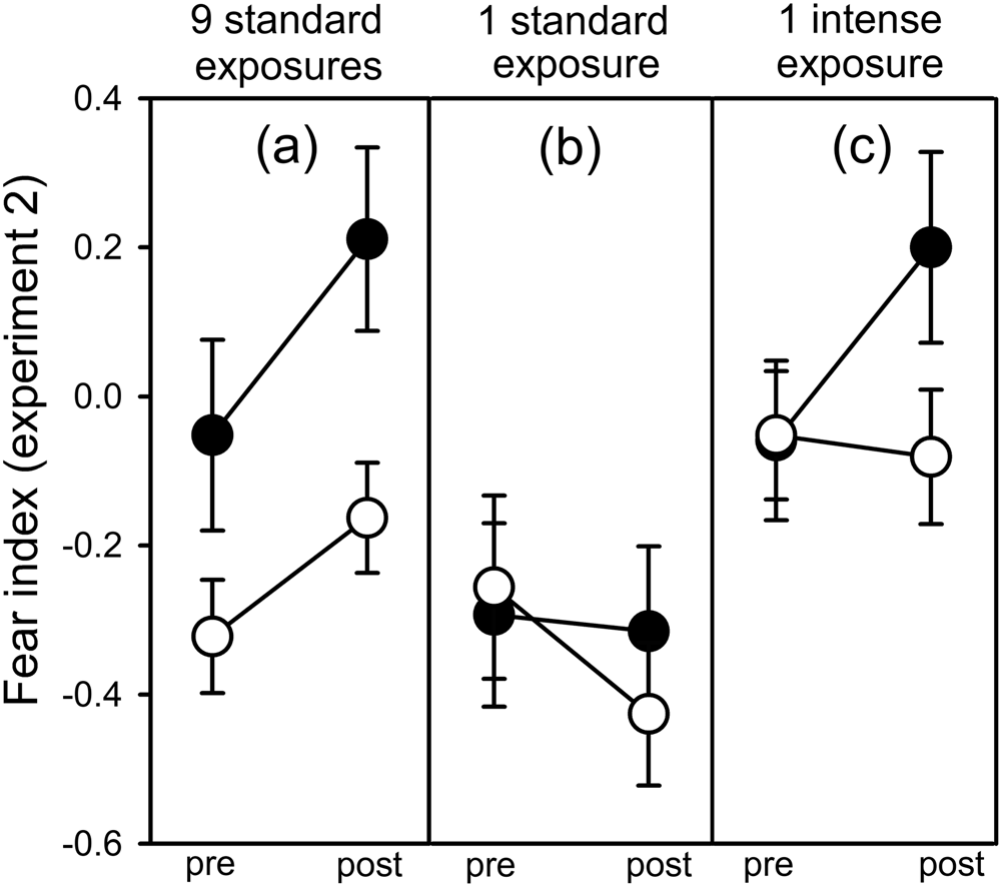
Mean (±SE) fear index from experiment 2 (re-triggered fear). Guppies were tested before (pre) and after (post) injection of a novel odour 8 days after exposure to background predation risk via alarm cues [either 9 standard exposures (**a**), 1 standard exposure (**b**), or 1 intense exposure that was 9× higher than the standard exposure (**c**)] and 1 day following the re-exposure treatment with the standard concentration of alarm cues (black circles) or water (white circles). See methods for exposure details.

In experiment 2, we again found multiple significant interactions involving each of the fixed factors (Table 2). For the 9-standard-exposures group, post-hoc testing revealed a significant main effect of time (time: *F*_1, 28_ = 12.63, *p* = 0.001; time × test cue: *F*_1, 28_ = 0.76, *p* = 0.39; Table S3; Fig. 3a), thus indicating that guppies had retained their neophobic response over the 8-day period. However, there was also a significant main effect of the re-exposure treatment where the alarm cue re-exposure elevated the fear response (re-exposure: *F*_1, 32_ = 17.09, *p* = 0.020; Table S3; Fig. 3a). Again, the 1-standard-exposure group was not neophobic and was not induced into fearful behaviour from the re-exposure treatment (all fixed factors: *p* > 0.20; Fig. 3b; Table S3). In contrast, the 1-intense-exposure group was no longer neophobic unless they were re-exposed to the standard concentration of alarm cues (i.e., re-triggered neophobia) (time × re-exposure: *F*_1, 28_ = 6.82, *p* = 0.014; Fig. 3c; Table 2). Their initially elevated baseline fear appeared to persist throughout the 8-day period, but this response was also no longer significant (background risk: *F*_2, 6.9_ = 2.44, *p* = 0.16; Fig. 3c; Table S4), although statistical power was relatively low for this comparison involving only water re-exposed individuals.

**Table 2:** Overall GLMM for experiment 2.

	*F*	*df*	*p*
Time	4.08	1, 84	0.047
Background risk	5.90	2, 14.3	0.014
Re-exposure	4.27	1, 14.3	0.057
Time × background risk	5.74	2, 84	**0.005**
Time × re-exposure	0.57	1, 84	**0.020**
Background risk × re-exposure	1.09	2, 14.3	0.36
Time × background risk × re-exposure	0.54	2, 84	0.58
Background pail	1.02	18, 66	0.45
Subject	4.36	66, 84	<0.001

Output for testing the fixed effects of time (pre/post stimulus), the background risk treatment (9  standard exposures, 1 standard exposure, 1 intense exposure), the re-exposure treatment (alarm cue or water), and their interactions, on the fear index 1 day after the re-exposure treatment (8 days after the background risk treatment), with the background pail and the subject as random factors. Significant terms of interest are in bold type.

## Discussion

We found evidence that a single dangerous event can induce baseline fear and neophobia for guppies when the magnitude of the risk is intense. This effect of risk intensity may explain the different patterns of induced neophobia observed in previous studies involving only a single exposure to alarm cues^35,41^. In this study, the single intense exposure caused a large overall response (both higher baseline fear and neophobia), but it was no longer significant after 8 days post risk, unlike the retention in the multiple exposures treatment. This suggests that the length of fear retention does not always correlate with the intensity of the initial response, and thus the single risk exposure was not a strong predictor of future risk. However, after the initial response waned, the subsequent re-exposure to the low-level threat re-triggered neophobia, despite that low-level threat being insufficient to induce fear behaviour in the absence of high background risk. Hence, neophobia had become easier to induce following the intense background experience. The single background exposure to the low-level threat did not induce fear behaviour, but repeated exposures to the low-level threat did. In this case, the induced neophobic response remained significant after 8 days, suggesting that multiple exposures influenced guppies to expect future threats, with the single re-exposure to the low-level threat elevating this retained neophobia.

Guppies in this study were exposed to predation risk at multiple time points, with 7 days separating the final risk exposure from the prior risk period. One treatment group transitioned from a high-infrequent threat to a low-infrequent threat, another from low-frequent to low-infrequent, and the other remaining consistent at low-infrequent risk. There is evidence that some species, such as wood frog tadpoles, *Lithobates sylvaticus*, can project trends in risk into their future responses^42,43^, but we saw no such ‘extrapolation’ in this study. Instead of projecting a trend of decreasing risk, the low-level re-exposure caused guppies to show re-triggered fear that matched the intensity of their initial fear. One explanation for the different pattern is that, in the aforementioned studies, risk transitioned from low to high (opposite of this study). Because the cost of under-responding to risk is so much greater than over-responding^44^, prey may be less likely to extrapolate from decreasing trends in risk. Another explanation is that species that experience major changes in the composition of predators throughout their lives will be more likely to project risk trends (e.g., the tadpoles in previous studies). Finally, the timing of events may, again, be an important factor. In one study on fear extinction in rats, gradually reducing risk, rather than abruptly, prevented the return of fear^45^.

Because fear neural pathways have been highly conserved across vertebrate taxa, animals exposed to risk are being used as models to explore dimensions of post-traumatic stress in humans^34,46,47^. Rats and mice are commonly used and have shown a high degree of model validity, particularly when exposed to predation risk relative to other types of stressors^46^. There is a wealth of literature on fishes exposed to predation risk, with several recent studies making a case for their validity in such applications, including for treatment with anxiolytic medication^32,48,49^. Yehuda and Antelman^50^ put forth a classic set of criteria that are specific to the validity of PTSD models. For each criterion, there are studies on fishes that have demonstrated parallels, with this study being an example of a brief one-time event inducing analogous behavioural changes (Table 3). Fishes may be a useful model for such research because their abundance can facilitate multi-factorial experiments that test source-treatment interactions with statistical reliability from large sample sizes. Moreover, combining knowledge obtained from multiple animals models, including new models such fish, may help us, as Borghans and Homberg^34^ stated, to obtain the ‘optimal reflection’ of PTSD.

**Table 3:** Behavioural research on fishes in comparison to Yehuda and Antelman’s (1993) criteria for evaluation of animal models for PTSD.

Criterion	Support in fishes	Example literature
Even brief stressors induce effects	A single exposure to intense risk can induce baseline fear behaviour and neophobia.	Abudayah & Mathis^35^; Current study
Intensity-dependent responses	Higher background risk induces more intense neophobia.	Brown *et al*.^30^; Brown *et al*. ^51^
Persistence of alterations over time	Induced neophobia can last for weeks, and likely longer with more intense risk.	Brown *et al*.^31^; Joyce et al.^12^
Bi-directional expression of changes	Activity can decrease (freezing behaviour) and increase (pacing).	Crane & Ferrari^52^; Crane *et al*.^53^
Reliable inter-individual variability	High inter-individual variability in fear reactions is common.	Bell & Sih^54^; Brown *et al*.^10^

Our study appears to be the first on re-triggered fear in a fish species. This phenomenon has previously been reported among rats and humans^17^), and we suspect that it is widespread, at least across vertebrate taxa due to similar fear neural pathways^47^. While previous studies have focused on the cognitive process involved in the return of fear, its ecological/functional role has received less attention. In the natural world, fear reactions help prey survive encounters with predators^11,51,52^. However, environments are not static and may transition between periods of high predation threat and low threat. When environmental change occurs rapidly^53^, prey will need time to recognize and adjust to the new conditions. If a risky environment suddenly becomes safe, continued fear responses (i.e., false positives in the context of Error Management Theory) become costly and should cease^2,44^. The speed at which this occurs should relate to the intensity of the original threat, but perhaps more so, to the likelihood that the predation threat will continue to return. For instance, prey that are naïve to risk in their environment might interpret a single risky event as being an isolated incident and not a predictor of future risk. Thus, they should not maintain heightened vigilance once they recognize that the threat is gone. In contrast, prey that have experienced risk multiple times in the past should expect that it will return, and when it returns, to occur repeatedly. Likewise, if a previous experience with risk was intense, prey would know any new sign of risk could be associated with an intense threat. In both cases, heightened vigilance should be maintained until a substantial amount of safety information (e.g., time without risk) has been obtained, as non-responses when danger still exists (i.e., false negatives) are generally the most costly mistakes (resulting in injury or death). After eventually waning, however, the first new sign of any danger should cause prey to quickly prepare themselves for the possibility of a re-occurring or more severe threat, which will increase their chance of survival if their past experience accurately reflected future risk patterns. Hence, in such an ecological scenario, which is likely common in fluctuating environments, an easily re-triggered state of predation fear is an adaptive response.

## Methods

### Fish collection, maintenance, and cues

All methods were carried out in accordance with relevant guidelines and regulations. Using a seine net, we collected adult female guppies (20–35 mm total length) from sites on the upper portion of the Aripo River in the Northern Range of the Republic of Trinidad and Tobago. The sites are considered as ‘low-risk’ for guppies because they lack aquatic predators of adult guppies^54–56^. The guppies from these sites are not fearful toward novel odours under natural conditions, but repeated exposure to alarm cues induces neophobic behaviour^30,40^. After collection, we transported guppies to the laboratory at the University of the West Indies, St. Augustine (23 °C and a 12:12 L:D cycle), holding them in a 250-L glass aquaria filled with 185 L of dechlorinated tap water (hereafter, water) which was filtered and aerated (ISTA BioSponge filters). Guppies were fed twice daily with flake food (Omega One, Freshwater Fish) throughout the experiment.

Following previously established methods^57,58^, we used 100 donor individuals to obtain a solution of alarm cues that was sufficient for multiple experiments conducted in 2019, including the experiments presented here. First, the donors were euthanized by cervical dislocation and decapitation. The head, tail, and visceral contents were discarded, and the carcasses were sized and homogenized in water at a concentration of 0.1 cm^2^ tissue per mL. The cues were then stored in 20-mL aliquots at −20 °C until use. For use as a novel odour in the experiment, we mixed a solution of 12 drops of lemon extract (Blanches) in 600 mL of water, as in previous studies on neophobic behaviour in guppies^30^.

### Background risk treatments

For the background risk phase of the experiment, we moved 192 guppies into 24 opaque pails (8 individuals per pail). The pails (7.5 L, 20 × 20 × 22 cm) were filled with 4.5 L of water and received aeration via air stones connected to air pumps. Each pail was assigned to one of three background risk treatments (Fig. 1): either 9 standard exposures to alarm cues, 1 standard exposure, or 1 intense exposure. A standard exposure consisted of gently injecting 3 mL of the alarm cue solution into the pail with a syringe resulting in a final concentration ~1 cm^2^ of skin per 15 L of water. For the 1-intense-exposure group, we used 27 mL of the alarm cue solution and thus the exposure was 9 times greater than the standard exposure (1 × 3 mL vs. 1 × 27 mL) and matched the total amount of alarm cue used in the 9-standard-exposures treatment (9 × 3 mL vs. 1 × 27 mL). Hence, this treatment simulated a predator that was consuming several guppies in close proximity (rare in our study population^54^). The exposure phase occurred over 3 days (3×/d) between 0800 and 1600 each day with >2 hours between exposures. The 9-standard-exposures group received alarm cues at each exposure, whereas the 1-standard-exposure and 1-intense-exposure groups received a water injection for the first 8 exposures and then an alarm cue injection on the final exposure. At the end of each day, we replaced 50% of the water in each pail with fresh water.

### Experiment 1 testing

For half of the guppies (12 of the 24 pails), behavioural testing occurred 1 day after the background risk period (Fig. 1). An hour before testing, these guppies were randomly assigned and moved into individual test tanks (22 L, 45 × 21 × 23 cm) that contained 20 L of water and an airstone affixed to the back wall of the tank. Opaque barriers between tanks prevented guppies from observing adjacent tanks. A small amount of flake food was added 15 min before trials began. To introduce stimuli during the trials, each tank had a 1-m ‘injection hose’ attached to the airstone. Each trial consisted of a 3-min pre-stimulus observation (a blind personal observation) followed by the injection of the test stimulus and a 3-min post-stimulus observation. The test stimulus was 10 mL of either water or novel odour (Fig. 1). During both the pre- and post-stimulus periods, we recorded the occurrence of certain behaviours: dashing (rapid erratic darting), freezing (centre of body not moving), foraging (moving and striking at food), or calm swimming (not engaged in the other recorded behaviors or in stereotypic pacing behaviour)^59,60^ at 10-s intervals. We tested 15–17 guppies per treatment group. A few individuals jumped out of the tank during the acclimation period, and we gently returned these guppies to their tanks but did not test them.

### Re-exposure phase

Whereas guppies in half of the pails were tested 1 day after the background risk phase (experiment 1), guppies in the other half of pails simply remained in their background pails. Each day, guppies were fed and received water changes but were otherwise undisturbed for 6 days. Then on the 7^th^ day post-risk, the guppies received a single exposure to either the standard concentration of alarm cues or water (6 pails per treatment, 3 mL injection per pail) (Fig. 1).

### Experiment 2 testing

The day following the re-exposure treatment, behavioural observations were conducted as in experiment 1, except all guppies were tested with novel odour (i.e., no water testing cue in this experiment) (Fig. 1). Sample sizes were 15 per treatment group.

### Statistical analysis

First, we converted the data to proportions (i.e., the number of 10-s intervals performing a behaviour divided by the total number of intervals). Then, we calculated a single ‘fear index’ by subtracting the proportion of time spent in calmer behaviours (calm swimming and foraging) from the proportion of time spent in overt fear behaviours (dashing and freezing). Hence, the index ranged from −1 to 1, with higher scores representing increased fear. Parametric testing assumptions were met for this index. To draw conclusions about neophobia we analyzed differences among the treatments in the change between the pre- and post-stimulus data (i.e., differences in slopes). We also analyzed the pre-stimulus data alone to draw conclusions about differences in baseline behaviour.

For experiment 1 neophobia, we conducted a repeated-measures GLMM where the background risk treatment (9-standard-exposures, 1-standard-exposure, or 1-intense-exposure), the test cue (water or novel odour), and time (pre or post) were fixed factors and the background pail and subject were random nested factors (i.e., Type I sum of squares). For post-hoc analysis, we split the data by the background risk treatment and conducted separate GLMMs on each background risk group using only the test cue and time as fixed factors and the background pail and subject as random factors. For baseline fear, we used a simpler GLMM without time and test cue. The background risk treatment was a fixed factor and the background pail was a random nested factor. In this case for post-hoc testing, we repeated the analysis with the inclusion of specific groups to make comparisons to the negative control (9-standard-exposure vs. 1-intense-exposure and 1-intense-exposure vs. 1-standard-exposure), reducing α to 0.05/2 = 0.025 because the control group was used twice in post-hoc testing.

For data from experiment 2, we used the same approach as in experiment 1. The repeated-measures GLMM for neophobia included the same terms except the re-exposure treatment was used as a fixed factor rather than the test cue (only 1 test cue in experiment 2). For baseline behaviour, the model was identical to that used in experiment 1 but included only the individuals that did not receive a re-exposure to alarm cues (i.e., only the water re-exposure group), as this may have affected their baseline behaviour. All analyses were conducted in SPSS 23.

### Ethics approval

This research was approved by the Concordia University Animal Research Ethics Committee (protocol # AREC30000255). Guppies were collected under a permit issued by the Republic of Trinidad and Tobago’s Ministry of Agriculture, Land and Marine Resources.

## Supplementary information

Supplementary Information.

Supplementary Information 2.

## Data Availability

Data and additional statistical tables are provided as electronic supplementary material.
