# Supplementary material for: The propensity for re-triggered predation fear in a prey fish
Source: Sci Rep. 2020 Jun 9;10:9253. doi: 10.1038/s41598-020-65735-1 (PMC7283299; doi:10.1038/s41598-020-65735-1)
Supplement: Supplementary file 1 — Supplementary Information. [file 41598_2020_65735_MOESM1_ESM.docx]

*Supplementary Information for:*

**The propensity for re-triggered predation fear in a prey fish**

Adam L. Crane^1^*, Laurence E.A. Feyten^1^, Indar W. Ramnarine^2^ & Grant E. Brown^1^

^1^Department of Biology, Concordia University, Montreal, Canada

^2^Department of Life Sciences, University of the West Indies, St. Augustine, Trinidad and Tobago

*Corresponding author email: [adam.crane@concordia.ca](mailto:adam.crane@concordia.ca)

**Table S1.** **Experiment 1 post-hoc GLMMs.** The effects of two fixed factors [time (pre or post-stimulus period) and test cue (novel odour or water)] and their interaction on the fear index 1 day after background risk, with the background pail and the subject as random factors (repeated-measures nested designs with Type I sum of squares). Bold type and asterisks represent significant terms of interest.

| **Background risk** | **Model term** | ***F*** |  | ***df*** |  | ***p*** |
| --- | --- | --- | --- | --- | --- | --- |
| 9 standard exposures | Time | 13.55 |  | 1, 32 |  | 0.001 |
|  | Test cue | 3.35 |  | 1, 29.7 |  | 0.077 |
|  | Time × test cue | 14.80 |  | 1, 32 |  | **0.001*** |
|  | Background pail | 0.80 |  | 7, 25 |  | 0.59 |
|  | Subject | 6.22 |  | 25, 32 |  | <0.001 |
| 1 standard exposure | Time | 0.33 |  | 1, 29 |  | 0.57 |
|  | Test cue | <0.01 |  | 1, 32 |  | 0.98 |
|  | Time × test cue | 3.20 |  | 1, 29 |  | 0.084 |
|  | Background pail | 0.23 |  | 7, 22 |  | 0.97 |
|  | Subject | 16.48 |  | 22, 29 |  | <0.001 |
| 1 intense exposure | Time | 5.82 |  | 1, 32 |  | 0.022 |
|  | Test cue | 0.31 |  | 1, 26.8 |  | 0.58 |
|  | Time × test cue | 4.47 |  | 1, 32 |  | **<0.042*** |
|  | Background pail | 0.26 |  | 7, 25 |  | 0.97 |
|  | Subject | 9.58 |  | 25, 32 |  | <0.001 |

**Table S2. Experiment 1 post-hoc GLMM.** Output for testing the fixed effect of the background risk treatment [9 standard exposures (9SE), 1 standard exposure (1SE), or 1 intense exposure (1IE)] on the pre-stimulus baseline fear index, with the background pail as a random factor. Significant terms of interest are in bold type.

|  | ***F*** |  | ***df*** |  | ***p*** |  |
| --- | --- | --- | --- | --- | --- | --- |
| *Overall model* |  |  |  |  |  |  |
| Background risk | 4.69 |  | 2, 20.1 |  | **0.021*** |  |
| Background pail | 0.47 |  | 21, 75 |  | 0.98 |  |
| *Post-hoc 9SE vs. 1SE* |  |  |  |  |  |  |
| Background risk | 0.06 |  | 1, 13.6 |  | 0.82 |  |
| Background pail | 0.54 |  | 14, 49 |  | 0.89 |  |
| *Post-hoc 1IE vs. 1SE* |  |  |  |  |  |  |
| Background risk | 7.97 |  | 1, 13.4 |  | **0.014*** |  |
| Background pail | 0.36 |  | 14, 49 |  | 0.98 |  |

**Table S3. Experiment 2 post-hoc GLMMs.** The effects of two fixed factors [time (pre or post-stimulus period) and the re-exposure treatment (alarm cues or water)] and their interaction on the fear index 1 day after background risk, with the background pail and the subject as random factors (repeated-measures nested designs with Type I sum of squares). Bold type and asterisks represent significant terms of interest.

| **Background risk** | **Model term** | ***F*** |  | ***df*** |  | ***p*** |
| --- | --- | --- | --- | --- | --- | --- |
| 9 standard exposures | Time | 12.63 |  | 1, 28 |  | **0.001** |
|  | Re-exposure | 17.09 |  | 1, 3.4 |  | **0.020*** |
|  | Time × re-exposure | 0.76 |  | 1, 28 |  | 0.39 |
|  | Background pail | 0.35 |  | 6, 22 |  | 0.90 |
|  | Subject | 5.89 |  | 22, 28 |  | <0.001 |
| 1 standard exposure | Time | 1.47 |  | 1, 28 |  | 0.24 |
|  | Re-exposure | 0.03 |  | 1, 5.5 |  | 0.87 |
|  | Time × re-exposure | 0.87 |  | 1, 28 |  | 0.36 |
|  | Background pail | 2.85 |  | 6, 22 |  | 0.033 |
|  | Subject | 2.27 |  | 22, 28 |  | 0.021 |
| 1 intense exposure | Time | 4.31 |  | 1, 28 |  | 0.047 |
|  | Re-exposure | 2.43 |  | 1, 3.1 |  | 0.22 |
|  | Time × re-exposure | 6.82 |  | 1, 28 |  | **0.014*** |
|  | Background pail | 0.44 |  | 6, 22 |  | 0.84 |
|  | Subject | 6.91 |  | 22, 28 |  | <0.001 |

**Table S4. Experiment 2 post-hoc GLMM.** Output for testing the fixed effect of the background risk treatment on the pre-stimulus baseline fear index for water re-exposed individuals, with the background pail as a random factor. Significant terms of interest are in bold type.

|  | ***F*** |  | ***df*** |  | ***p*** |  |
| --- | --- | --- | --- | --- | --- | --- |
| *Overall model* |  |  |  |  |  |  |
| Background risk | 2.44 |  | 2, 6.9 |  | 0.16 |  |
| Background pail | 0.84 |  | 9, 33 |  | 0.52 |  |
